# Impact of endometriotic cystectomy on ovarian reserve function and ovulation induction outcomes in women with endometriosis undergoing assisted reproductive technology

**DOI:** 10.3389/fendo.2025.1687765

**Published:** 2026-01-19

**Authors:** Yutao Li, Yu Gong, Haiyan Jiang, Meng Ji

**Affiliations:** 1 Department of Assisted Reproduction Center, Sichuan Provincial People’s Hospital, University of Electronic Science and Technology of China, Chengdu, Sichuan, China; 2 Department of Outpatient, Sichuan Provincial People’s Hospital, University of Electronic Science and Technology of China, Chengdu, Sichuan, China

**Keywords:** endometriosis, endometriotic cystectomy, IVF/ICSI, ovarian reserve function, ovulation induction results

## Abstract

**Background:**

This study aims to evaluate the impact of endometriotic cysts and prior ovarian endometriotic cystectomy on ovarian reserve function in women with endometriosis undergoing assisted reproductive technology.

**Methods:**

In this retrospective cohort study, 3,517 endometriosis patients receiving *in vitro* fertilization/intracytoplasmic sperm injection (IVF/ICSI) between January 2016 and April 2022 were reviewed. Participants were stratified into three groups: Group A (control, n=494): patients without endometriosis undergoing IVF/ICSI for male factor infertility; Group B (non-surgical, n=217): endometriosis patients with untreated endometriotic cysts; Group C (surgical, n=122): endometriosis patients with prior cystectomy. Antral follicle count (AFC), follicle-stimulating hormone (FSH), anti-Müllerian hormone (AMH), gonadotropin (Gn) dose, number of retrieved oocytes, number of mature metaphase II (MII) oocytes and the proportion of patients with diminished ovarian reserve (DOR; AMH<1.1ng/ml) were compared across groups.

**Results:**

Ovarian reserve markers were highest in Group A [AMH: 2.88 (1.64–4.45) ng/mL; AFC: 13 (8.5–17)], followed by Group B [AMH: 2.70 (1.59–4.05) ng/mL; AFC: 11 (7–16)], with both significantly exceeding Group C [AMH: 1.97 (1.02–3.05) ng/mL; AFC: 10 (4–15)] (all P < 0.01). The incidence of DOR was significantly higher in Group C (26.23%) than in Group A (13.56%) and Group B (12.90%) (P < 0.05). The total Gn dose was significantly higher in Groups B and C than in Group A. The number of retrieved oocytes and MII oocytes did not differ significantly between Groups A and B, but both were significantly higher than in Group C (P < 0.01).

**Conclusions:**

A history of endometriotic cystectomy is associated with significantly diminished ovarian reserve and poorer ovarian response during controlled ovarian stimulation. These findings highlight the importance of individualized surgical decision-making for reproductive-aged women with endometriomas, weighing potential benefits against the risk of iatrogenic damage to ovarian function.

## Introduction

Endometriosis, a common chronic estrogen-dependent gynecological disorder affecting 10% of reproductive-aged women globally. It is characterized by the presence of endometrial tissue outside the uterine cavity (1). The etiology of endometriosis is multifactorial, involving genetic predisposition, metabolic and microbiomic influences, immune dysfunction, endocrine disruptions, and environmental factors (2–4). The incidence rate of endometriosis among women of childbearing age is 2% - 10%, rising to 20%–50% in infertile populations (5–8). Ovarian endometrioma (OMA) is the most common manifestation of endometriosis, accounting for 17% - 44% of endometriosis (9).

The management of OMA presents a persistent clinical challenge. While medical management effectively alleviates pain symptoms by suppressing estrogen production (10), it doesn’t address the underlying pathology or improve fertility outcomes. Surgical cystectomy, specifically laparoscopic cystectomy, remains the gold standard for the definitive removal of endometriotic cysts (5, 11). However, its use is contentious due to the significant risk of iatrogenic injury to the ovarian reserve. Systematic reviews demonstrate that laparoscopic cystectomy reduces anti-Müllerian hormone (AMH) levels by 38% postoperatively (12), with bilateral cystectomy correlating with a 2.9-fold increased risk of premature ovarian insufficiency (13). Furthermore, 54% of patients require reoperation within 5 years due to recurrence, compounding cumulative ovarian damage (14).

Preserving ovarian reserve is paramount, as it directly determines reproductive potential and predicts outcomes in assisted reproductive technologies. A critical unresolved question in the field is the relative contribution of the OMA itself versus the surgical intervention to ovarian impairment. Although existing evidence confirms cystectomy-induced ovarian reserve depletion, whether OMA per se contributes to diminished ovarian reserve (DOR) through chronic inflammation-mediated follicle apoptosis remains unelucidated (15). Notably, several meta-analyses have shown that, compared to women without OMA, those with endometriomas yield significantly fewer oocytes yet show no significant differences in gonadotropin consumption, embryological outcomes, clinical pregnancy, or live birth rates (16–18). In addition, multiple systematic reviews have concluded that performing cystectomy before *in vitro* fertilization/intracytoplasmic sperm injection (IVF/ICSI) does not improve treatment outcomes (16, 17, 19). A recent retrospective study demonstrates that advanced endometriosis negatively affects the cumulative clinical pregnancy rate per oocyte retrieval cycle, which may be attributed to poor ovarian response associated with OMA themselves or their surgical removal; however, the precise underlying mechanism remains unclear (20). Cumulatively, these observations call for a cautious, evidence-based evaluation of the risk-benefit profile of endometrioma surgery in women pursuing IVF/ICSI.

Therefore, this study aimed to comprehensively investigate the influence of ovarian endometriotic cystectomy on the ovarian reserve function of patients with endometriosis, as well as its impact on the ovulation induction results during fertility treatments. By comparing different groups of patients with or without a history of cystectomy and a control group without endometriosis, we hope to provide valuable insights for clinicians and patients when considering treatment options and evaluating potential reproductive outcomes.

## Methods

### Patients

This retrospective cohort study initially comprised 14,871 consecutive couples undergoing IVF/ICSI cycles at two assisted reproductive centers between January 2016 and April 2022. The study group was identified based on a diagnosis of endometriosis. The exclusion criteria were: (1) age <20 or ≥50 years; (2) other gynecological diseases that may affect the study results, such as polycystic ovary syndrome, or systemic diseases; (3) history of pelvic radiotherapy or chemotherapy or other pelvic surgeries in the past; (4) congenital reproductive system malformations; (5) known chromosomal abnormalities; (6) mental disorders or inability to cooperate with the study protocol;(7) patients receiving any concomitant medical therapy (e.g., GnRH agonists, oral contraceptives) specifically for endometriosis during or within 3 months before the IVF/ICSI cycle. In order to study the impact of previous excision of endometriotic cysts on ovarian reserve function and ovulation induction results, we subdivided the endometriosis group into two subgroups: Group B (non-surgical): endometriosis patients with untreated endometriotic cysts; Group C (surgical): endometriosis patients had undergone excision of endometriotic cysts previously.

We also selected patients who underwent IVF/ICSI treatment solely for male factor infertility, with no female factor infertility, during the same period as the control group (Group A). Controls were matched to the study group based on age (within 1 year) and body mass index (BMI) (within 1 kg/m²). This retrospective study was approved by the Reproductive Medicine Ethics Committee of Sichuan Provincial People’s Hospital (Approval number: 20241101).

All patients’ basic information, including age, BMI, duration of infertility, type of infertility, and causes of infertility, were collected from the patient’s electronic medical record system on March 1, 2025. Patients with missing or incomplete data regarding ovarian reserve markers or surgical history were excluded to ensure data integrity.

### Measures of ovarian reserve

Ovarian reserve was assessed using biochemical tests and AFC. Biochemical tests of ovarian reserve including measurement of follicle-stimulating hormone (FSH), luteinizing hormone (LH) and AMH on day 2, or 3 of the menstrual cycle. FSH, LH, and AMH were measured by electrochemiluminescence (Roche, Switzerland), with detection limits of 0.1 mIU/mL for FSH, 0.1 mIU/mL for LH, and 0.01 ng/mL for AMH. Inter- and intra-assay coefficients of variation for AMH, FSH, and LH were all <5%, as per the manufacturer’s specifications. AFC, defined as the total number of follicles measuring 2–10 mm in diameter observed on day 2 or 3 via transvaginal ultrasonography, was performed by experienced sonographers to ensure consistency. The primary criterion for DOR was AMH <1.1 ng/ml. Although FSH>10 IU/L and AFC <5–7 are also commonly used, AMH was selected as the primary marker due to its higher sensitivity in endometriosis patients, particularly post-surgery (21, 22).

### Controlled ovarian hyperstimulation protocol

All patients received a routine ovulation induction protocol. Ovarian stimulation was performed using human menopausal gonadotropin (HMG), recombinant FSH, or urinary FSH. The starting Gn dose was determined based on the woman’s age, BMI, AFC, AMH and previous treatment response. Ovarian response was monitored via serum Estradiol (E2) concentrations and ultrasound from stimulation day 5 or 6 onward. When at least three follicles reached ≥17 mm or two follicles reached ≥18 mm in diameter, a single dose of human chorionic gonadotropin (HCG) ranging from 5,000 to 10,000 IU or recombinant HCG 250µg was administered. Oocyte retrieval was performed approximately 36 hours later under ultrasound-guided transvaginal aspiration.

### Statistics

Using SPSS 27.0 statistical software for statistical analysis; The Kolmogorov-Smirnov test was used to test the normality of metric data. Non-normal distribution data or ordinal data were presented by median (P25, P75), and group comparisons were analyzed using the Kruskal-Wallis H rank sum test, with pairwise comparisons conducted using the Bonferroni method. Count data were presented by frequency and percentage (n, %), and the comparison of rates between groups was conducted using the chi-square test. A two-tailed P < 0.05 was considered statistically significant. A *post-hoc* power analysis indicated that the sample size provided 80% power to detect a moderate effect size (d = 0.4) in AMH levels between groups at α = 0.05.

## Results

**Figure 1:**
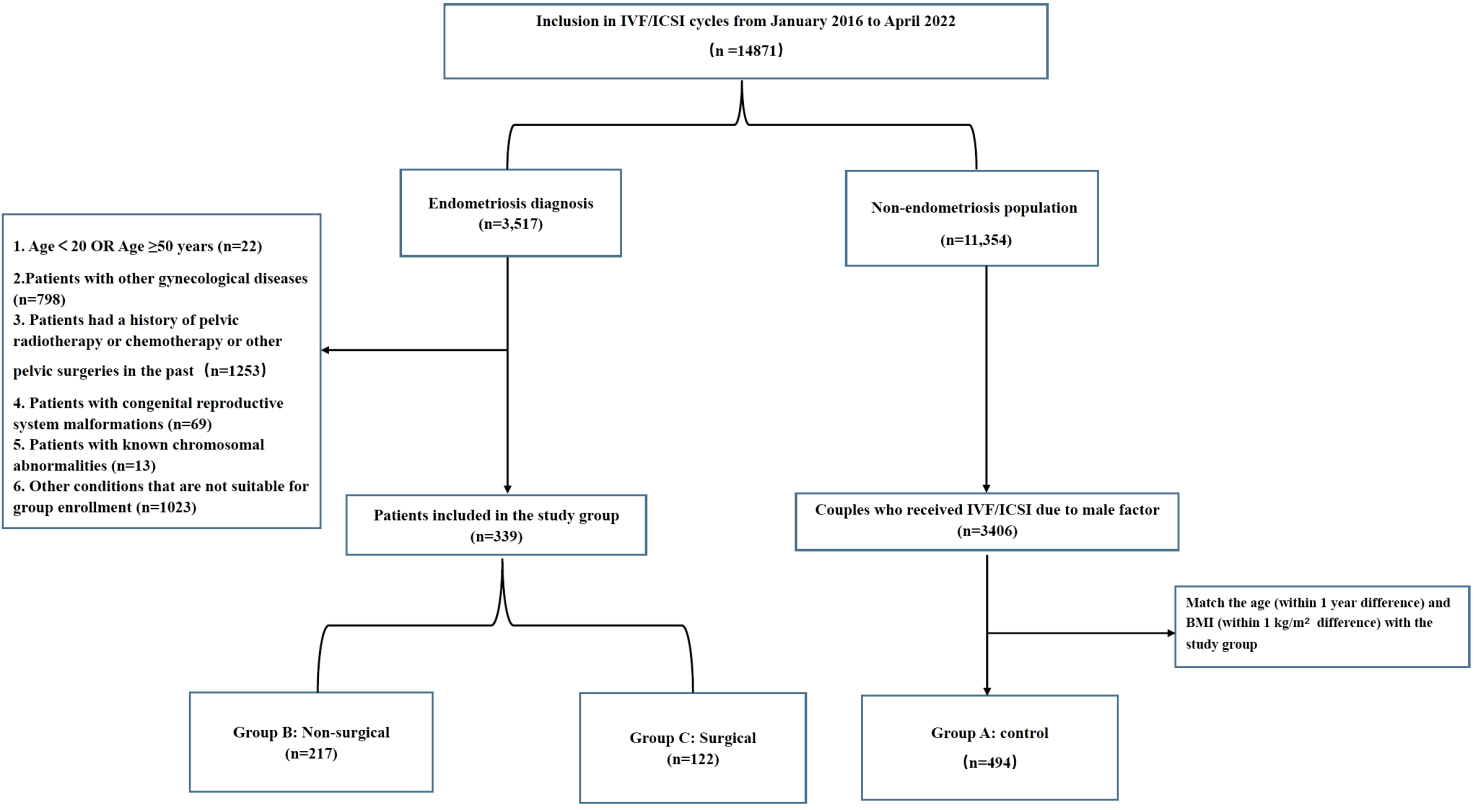
The flow chart of the inclusion and exclusion process.

A total of 3,517 medical records of female patients with endometriosis were reviewed. After applying the exclusion criteria, 339 cases with current or previous endometriotic cysts were included: 217 with endometriotic cysts (Group B) and 122 with a history of endometriotic cystectomy (Group C). Meanwhile, 494 patients who underwent IVF/ICSI due to male factors, matched for age and BMI with the study group, were selected as controls (Group A). A flow chart of the inclusion process is shown in **Figure 1**.

Baseline characteristics of the groups are shown in **Table 1**. Maternal age and BMI were similar across groups due to matching. The frequency of primary infertility was also comparable. In terms of infertility duration, Group A had the longest duration, followed by Group C and then Group B, with statistically significant differences.

**Table 1:** Baseline clinical characteristics of different groups.

Parameter	Group A (n=494)	Group B (n=217)	Group C (n=122)	*P*
Age (years)	30.91 ± 5.13	31.06 ± 3.68	30.92 ± 4.05	0.914
BMI (kg/m^2^)	21.21 ± 2.60	20.95 ± 2.46	21.17 ± 2.71	0.444
Duration of infertility (years)	4(2,7) ^a^	2(1,4) ^b^	3(2,5) ^c^	<0.001
Types of infertility				0.273
Proportion of secondary infertility (%)	360(72.9%)	161(74.2%)	81(66.4%)	
Proportion of primary infertility (%)	134(27.1%)	56(25.8%)	41(33.6%)	

BMI, body mass index; Different letters (abc) on the same line indicate statistically significant differences between groups, with a P value < 0.05.

**Table 2** presents the comparisons of ovarian reserve function and ovulation induction outcomes. AFC showed a marked decreasing trend from Group A to Group C (p < 0.001). AMH levels in Groups A and B were significantly higher than in Group C, but no significant difference was observed between Groups A and B. FSH levels did not differ significantly between Group A and Groups B and C, though FSH in Group C was significantly higher than in Group B. Ovarian reserve function was significantly lower in the cystectomy group (Group C), as reflected by lower AFC, lower AMH, and higher FSH. The total amount of Gn dose was significantly higher in Groups B and C than in Group A, but no significant difference was found between Groups B and C. The numbers of retrieved oocytes and MII oocytes were significantly higher in Groups A and B than in Group C, with no significant difference between Groups A and B.

**Table 2:** Ovarian reserve and controlled ovarian hyperstimulation outcomes of different groups.

Parameter	Group A (n=494)	Group B (n=217)	Group C (n=122)	*P*
No. of AFC	13(8.5,17)^a^	11(7,16)^b^	10(4,15)^c^	<0.001
FSH (IU/L)	7.69(6.57,9.1)^a,b^	7.38(6.18,9.24)^b^	8.39(6.81,10.27)^a^	0.007
AMH (ng/ml)	2.88(1.64,4.45)^a^	2.7(1.59,4.05)^a^	1.97(1.02,3.05)^b^	<0.001
Total dose of GN used (IU)	1800(1350,2250)^b^	1875(1500,2325)^a^	2025(1350,2475)^a^	0.003
No. of oocytes retrieved	9(4,14)^a^	8(5,14)^a^	6(3,10)^b^	<0.001
No. of MII eggs	9(4,13)^a^	8(4,12)^a^	6(2.75,9)^b^	<0.001

AFC, antral follicle count; FSH, follicle-stimulating hormone; AMH, anti-mullerian hormone; Gn, Gonadotropins; MII, mature. Different letters (abc) on the same line indicate statistically significant differences between groups, with a P value <.

**Figure 2:**
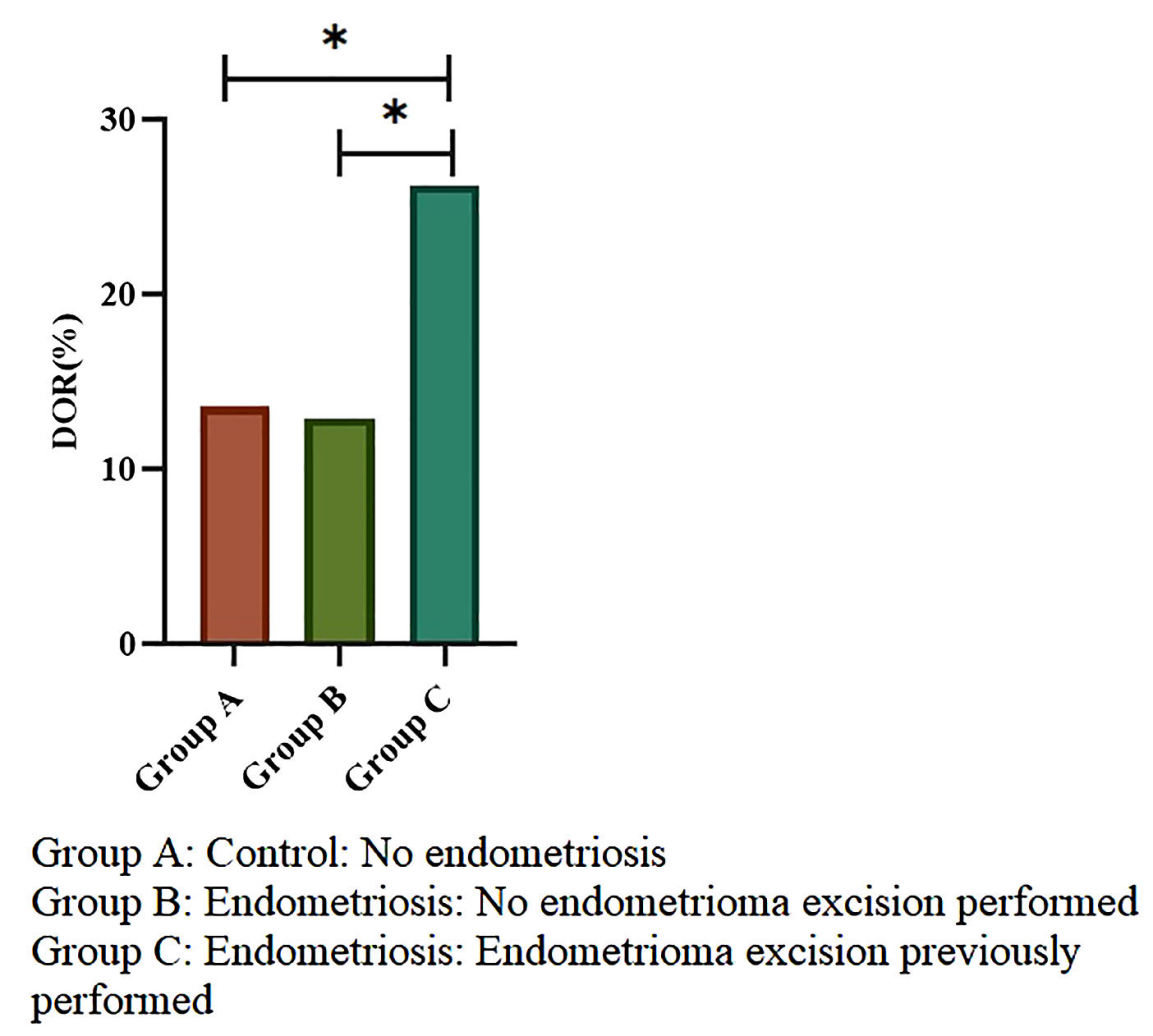
Incidence of diminished ovarian reserve across groups. Group A: n=494, Group B: n=217, Group C: n=122. Chi-square test was used; *indicates p < 0.05. Error bars represent 95% confidence intervals.

**Figure 2** shows the incidence of DOR in the three groups. The incidence was significantly lower in Groups A and B than in Group C (13.56% and 12.9% vs. 26.23%). The relative risk of DOR in Group C was 1.93 (95% CI: 1.32–2.83) compared to Group A and 2.03 (95% CI: 1.29–3.21) compared to Group B. No significant difference was detected between Group A and Group B.

## Discussion

This study indicates that a history of endometriotic cystectomy is associated with markers of decreased ovarian function, including lower AMH levels, reduced AFC, and a higher incidence of DOR, compared to both control and non-surgical endometriosis patients. Furthermore, the surgical group showed a profile of diminished ovarian response, characterized by higher Gn requirements and fewer retrieved and MII oocytes. Notably, the non-surgical endometriosis group showed only mild impairment in ovarian reserve compared to the control group, primarily reflected in reduced AFC.

Our findings carry a certain degree of persuasiveness. Firstly, it includes a relatively large sample size of endometriosis patients with clear stratification into surgical and non-surgical groups, alongside a carefully matched control group, enhancing the robustness of comparative analyses. Secondly, we employed a comprehensive set of ovarian reserve markers (AMH, AFC, FSH) and stimulation outcome measures, providing a multifaceted evaluation. Thirdly, by excluding patients on concurrent endometriosis medical therapy during the IVF cycle, we minimized a potential confounding factor, allowing for a clearer assessment of the isolated effects of cyst presence and prior surgery. Our results are consistent with existing literature demonstrating surgical damage to ovarian parenchyma during cystectomy (23–27). A recent meta-analysis of 36 studies comprehensively evaluated the impact of endometrioma surgery on ovarian reserve and found that surgery exerts a detrimental impact on AMH levels in the short, medium, and long - term with more pronounced declines in cases of bilateral lesions (28). This is likely because the surgical procedure damages ovarian tissue, disrupts the normal generation and development of follicles, reduces the number of recruitable and mature follicles, and consequently decreases the retrieval rate of mature oocytes. Specifically, cyst removal may directly damage the ovarian cortex and primordial follicles (29–31). In addition, surgery-induced local inflammation may lead to ovarian fibrosis, impairing blood supply and the follicular microenvironment, further compromising follicular developmental potential (32–34).

There is still no definite conclusion on whether endometriosis itself affects ovarian reserve function. Endometriosis itself may affect ovarian function through multiple pathways. Kitajima et al. found that ovaries with endometriomas, being more susceptible to local pelvic inflammation, exhibited enhanced follicular recruitment and early-stage follicular atresia (35). Pelvic adhesions and anatomical changes may also affect ovarian blood supply and function. The hypothesis that an endometrioma may impair ovarian reserve stems from concerns that the expanding cyst causes structural alterations and circulatory compromise, potentially reducing the primordial follicle pool (36). Consistent with this, a meta-analysis reported lower AMH levels in women with endometriomas compared to controls (37). However, this view is challenged by several studies (38–40). A large surgical cohort study found that endometriomas *per se* did not significantly lower AMH levels, whereas a history of cystectomy was independently associated with DOR (38). Another study suggested that significant AMH reduction is primarily observed in cases of bilateral endometrioma or larger cyst size, implying that the impairment may progress with disease severity (40). Interestingly, our study found that there were only minor differences in ovarian reserve between the non-surgical endometriosis group and the control group, mainly reflected in reduced AFC. Although AMH was lower in the endometriosis group, the difference was not significant. Moreover, although the endometriosis group required higher Gn doses, there were no significant differences in ovulation induction outcomes between the endometriosis and control groups. We speculate that the presence of endometriotic cysts may exert mechanical compression on the ovarian cortex, leading to a reduction in antral follicles and an increased demand for exogenous gonadotropins to achieve adequate follicular growth. However, it should be noted that our study may be subject to potential self-selection bias. Patients in the surgical group may have presented with more severe endometriosis—characterized by larger cyst size or longer disease duration—which itself could adversely affect ovarian reserve. This concern is supported by a clinical study which demonstrated that both endometrioma size and bilaterality are independently associated with reduced AMH levels, suggesting a detrimental effect of the disease *per se* on ovarian function (41). Therefore, the poorer ovarian outcomes observed in our surgical group may reflect a compounded effect of both the underlying disease severity and the surgical intervention. Due to the inherent limitations of our retrospective design, we cannot definitively disentangle these two factors. The continuous enlargement of endometriotic cysts with the progression of endometriosis inevitably exerts further adverse effects on ovarian function. However, it remains unclear whether this negative impact outweighs the detrimental effect of surgical cystectomy on ovarian reserve. Clarifying this comparison holds significant clinical importance for further refining the indications for surgical intervention in patients with OMA.

The ESHRE 2022 guideline states that laparoscopic surgery may be an option for symptomatic endometriosis patients who desire pregnancy (42). However, as with previous guidelines, there is no evidence supporting surgical resection of endometriosis lesions before medically assisted reproduction, and thus no recommendation can be made. This cautious stance is strongly supported by a recent systematic review and meta-analysis by Bourdon et al. (34), which specifically evaluated the impact of endometriosis surgery on IVF/ICSI outcomes. The researchers found no statistically significant differences in live birth rates or ongoing pregnancy rates between patients who underwent surgery before IVF/ICSI and those who received first-line IVF/ICSI. Importantly, when the analysis was restricted to high-quality studies, the live birth rate per cycle was significantly reduced in the surgery group (34). In contrast, another meta-analysis reported that the presence of endometrioma itself did not significantly reduce live birth rates in IVF/ICSI cycles (16). Based on the cumulative evidence, clinical management of endometriotic cysts requires a nuanced approach. Surgical cystectomy should not be routine prior to IVF but reserved for specific indications such as significant pain, large cysts that may complicate oocyte retrieval, or suspicion of malignancy. For women with reproductive goals, fertility counseling is paramount and must explicitly address the critical trade-off between potential symptom relief and the demonstrated risk of iatrogenic damage to ovarian reserve, underscoring that surgery does not improve and may even impair IVF outcomes (43). When surgery is unavoidable, prioritizing fertility-preservation is paramount. This includes utilizing surgeons with specialized expertise, employing ablation techniques, and opting for barbed suture for hemostasis for preserving ovarian reserve (11, 44–46). Postoperative monitoring of ovarian reserve is also recommended to guide subsequent reproductive planning. Although laparoscopic cystectomy remains the standard surgical treatment for endometriotic cysts, new strategies continue to emerge to minimize iatrogenic ovarian tissue damage. While sclerotherapy and ablation techniques may cause less damage to ovarian tissue, current studies have not demonstrated significant advantages over surgery (47, 48). Therefore, there remains a need to explore therapeutic approaches that have a lesser impact on ovarian reserve.

This study has several limitations. First, as noted earlier, data collection relied on previous medical records, which may be incomplete or inaccurate. Most notably, detailed information regarding cyst characteristics, such as size and bilaterality, specific surgical parameters, including the surgeon’s experience, surgical technique, and the staging and severity of endometriosis were not available. The absence of these data hinders a more nuanced analysis and may introduce unmeasured confounding, as patients in the surgical group likely had larger or more complex cysts. Second, this study focused on ovarian reserve and stimulation outcomes; data on pregnancy and live birth rates were not systematically available for the entire cohort, which limits the assessment of ultimate reproductive success. Third, the lack of systematic long-term follow-up data prevents an assessment of the trajectory of ovarian recovery or the ultimate impact on cumulative pregnancy and live birth rates. Finally, although we analyzed and adjusted for some confounding factors, other unrecognized or uncontrollable factors, such as lifestyle and genetics, may have influenced the results. Future research should design prospective, large-sample randomized controlled trials, strictly control confounding factors, and conduct long-term follow-up to further explore the impact of the surgical removal of endometriotic cysts on ovarian reserve function and provide a more reliable basis for clinical treatment.

## Conclusion

In summary, within the limitations of this retrospective study, our findings suggest that endometriotic cystectomy is associated with a negative impact on ovarian reserve function and ovulation induction outcomes. Surgical intervention not only reduces AMH levels and AFC but also appears to double the risk of DOR. Notably, the presence of endometriotic cysts *per se* appears to have a limited impact on ovarian function. These outcomes highlight the need for cautious and individualized surgical decision-making. Therefore, for women of childbearing age with endometriotic cysts who have fertility requirements, the decision to undergo surgery should be made with caution, taking into account cyst size, bilaterality, and preoperative ovarian reserve status.

## Data Availability

The original contributions presented in the study are included in the article/supplementary material. Further inquiries can be directed to the corresponding author.

## References

[1] Giudice LC . Clinical practice. Endometriosis. New Engl J Med. (2010) 362:2389–98. doi: 10.1056/NEJMcp1000274, PMID: 20573927 PMC3108065

[2] Alio L Angioni S Arena S Bartiromo L Bergamini V Berlanda N . Endometriosis: seeking optimal management in women approaching menopause. Climacteric. (2019) 22:329–38. doi: 10.1080/13697137.2018.1549213, PMID: 30628469

[3] Deiana D Gessa S Anardu M Daniilidis A Nappi L D’Alterio MN . Genetics of endometriosis: a comprehensive review. Gynecological Endocrinol. (2019) 35:553–58. doi: 10.1080/09513590.2019.1588244, PMID: 30909768

[4] Saunders PTK Horne AW . Endometriosis: Etiology, pathobiology, and therapeutic prospects. Cell. (2021) 184:2807–24. doi: 10.1016/j.cell.2021.04.041, PMID: 34048704

[5] Dunselman GA Vermeulen N Becker C Calhaz-Jorge C D’Hooghe T De Bie B . ESHRE guideline: management of women with endometriosis. Hum Reprod (Oxford England). (2014) 29:400–12. doi: 10.1093/humrep/det457, PMID: 24435778

[6] Fuldeore MJ Soliman AM . Prevalence and symptomatic burden of diagnosed endometriosis in the United States: national estimates from a cross-sectional survey of 59,411 women. Gynecologic obstetric Invest. (2017) 82:453–61. doi: 10.1159/000452660, PMID: 27820938

[7] Cramer DW Missmer SA . The epidemiology of endometriosis. Ann New York Acad Sci. (2002) 955:11–22; discussion 34-6, 396-406. doi: 10.1111/j.1749-6632.2002.tb02761.x, PMID: 11949940

[8] Meuleman C Vandenabeele B Fieuws S Spiessens C Timmerman D D’Hooghe T . High prevalence of endometriosis in infertile women with normal ovulation and normospermic partners. Fertility sterility. (2009) 92:68–74. doi: 10.1016/j.fertnstert.2008.04.056, PMID: 18684448

[9] Yu O Schulze-Rath R Grafton J Hansen K Scholes D Reed SD . Adenomyosis incidence, prevalence and treatment: United States population-based study 2006-2015. Am J obstetrics gynecology. (2020) 223:94.e1–94.e10. doi: 10.1016/j.ajog.2020.01.016, PMID: 31954156

[10] Brown J Pan A Hart RJ . Gonadotrophin-releasing hormone analogues for pain associated with endometriosis. Cochrane Database systematic Rev. (2010) 2010:Cd008475. doi: 10.1002/14651858.CD008475.pub2, PMID: 21154398 PMC7388859

[11] Riemma G De Franciscis P La Verde M Ravo M Fumiento P Fasulo DD . Impact of the hemostatic approach after laparoscopic endometrioma excision on ovarian reserve: Systematic review and network meta-analysis of randomized controlled trials. Int J gynaecology obstetrics. (2023) 162:222–32. doi: 10.1002/ijgo.14621, PMID: 36503998

[12] Raffi F Metwally M Amer S . The impact of excision of ovarian endometrioma on ovarian reserve: a systematic review and meta-analysis. J Clin Endocrinol Metab. (2012) 97:3146–54. doi: 10.1210/jc.2012-1558, PMID: 22723324

[13] Coccia ME Rizzello F Capezzuoli T Evangelisti P Cozzi C Petraglia F . Bilateral endometrioma excision: surgery-related damage to ovarian reserve. Reprod Sci (Thousand Oaks Calif.). (2019) 26:543–50. doi: 10.1177/1933719118777640, PMID: 29848225

[14] Vercellini P Matteis SDE Somigliana E Buggio L Frattaruolo MP Fedele L . Long-term adjuvant therapy for the prevention of postoperative endometrioma recurrence: a systematic review and meta-analysis. Acta obstetricia gynecologica Scandinavica. (2013) 92:8–16. doi: 10.1111/j.1600-0412.2012.01470.x, PMID: 22646295

[15] Máté G Bernstein LR Török AL . Endometriosis is a cause of infertility. Does reactive oxygen damage to gametes and embryos play a key role in the pathogenesis of infertility caused by endometriosis? Front Endocrinol. (2018) 9:725. doi: 10.3389/fendo.2018.00725, PMID: 30555421 PMC6281964

[16] Hamdan M Dunselman G Li TC Cheong Y . The impact of endometrioma on IVF/ICSI outcomes: a systematic review and meta-analysis. Hum Reprod Update. (2015) 21:809–25. doi: 10.1093/humupd/dmv035, PMID: 26168799

[17] Yang C Geng Y Li Y Chen C Gao Y . Impact of ovarian endometrioma on ovarian responsiveness and IVF: a systematic review and meta-analysis. Reprod biomedicine Online. (2015) 31:9–19. doi: 10.1016/j.rbmo.2015.03.005, PMID: 25982092

[18] Alshehre SM Narice BF Fenwick MA Metwally M . The impact of endometrioma on *in vitro* fertilisation/intra-cytoplasmic injection IVF/ICSI reproductive outcomes: a systematic review and meta-analysis. Arch gynecology obstetrics. (2021) 303:3–16. doi: 10.1007/s00404-020-05796-9, PMID: 32979078 PMC7854445

[19] Nickkho-Amiry M Savant R Majumder K Edi-O’sagie E Akhtar M . The effect of surgical management of endometrioma on the IVF/ICSI outcomes when compared with no treatment? A systematic review and meta-analysis. Arch gynecology obstetrics. (2018) 297:1043–57. doi: 10.1007/s00404-017-4640-1, PMID: 29344847 PMC5849664

[20] Li A Zhang J Kuang Y Yu C . Analysis of IVF/ICSI-FET outcomes in women with advanced endometriosis: influence on ovarian response and oocyte competence. Front Endocrinol. (2020) 11:427. doi: 10.3389/fendo.2020.00427, PMID: 32765424 PMC7380107

[21] Younis JS Taylor HS . The impact of ovarian endometrioma and endometriotic cystectomy on anti-Müllerian hormone, and antral follicle count: a contemporary critical appraisal of systematic reviews. Front Endocrinol. (2024) 15:1397279. doi: 10.3389/fendo.2024.1397279, PMID: 38800489 PMC11116636

[22] Torella M Riemma G De Franciscis P La Verde M Colacurci N . Serum anti-Müllerian hormone levels and risk of premature ovarian insufficiency in female childhood cancer survivors: systematic review and network meta-analysis. Cancers. (2021) 13. doi: 10.3390/cancers13246331, PMID: 34944951 PMC8699404

[23] Uncu G Kasapoglu I Ozerkan K Seyhan A Oral Yilmaztepe A Ata B . Prospective assessment of the impact of endometriomas and their removal on ovarian reserve and determinants of the rate of decline in ovarian reserve. Hum Reprod (Oxford England). (2013) 28:2140–5. doi: 10.1093/humrep/det123, PMID: 23624580

[24] Ozaki R Kumakiri J Tinelli A Grimbizis GF Kitade M Takeda S . Evaluation of factors predicting diminished ovarian reserve before and after laparoscopic cystectomy for ovarian endometriomas: a prospective cohort study. J Ovarian Res. (2016) 9:37. doi: 10.1186/s13048-016-0241-z, PMID: 27329142 PMC4915097

[25] Chen Y Pei H Chang Y Chen M Wang H Xie H . The impact of endometrioma and laparoscopic cystectomy on ovarian reserve and the exploration of related factors assessed by serum anti-Mullerian hormone: a prospective cohort study. J Ovarian Res. (2014) 7:108. doi: 10.1186/s13048-014-0108-0, PMID: 25424986 PMC4255637

[26] Urman B Alper E Yakin K Oktem O Aksoy S Alatas C . Removal of unilateral endometriomas is associated with immediate and sustained reduction in ovarian reserve. Reprod biomedicine Online. (2013) 27:212–6. doi: 10.1016/j.rbmo.2013.04.016, PMID: 23768623

[27] Goodman LR Goldberg JM Flyckt RL Gupta M Harwalker J Falcone T . Effect of surgery on ovarian reserve in women with endometriomas, endometriosis and controls. Am J obstetrics gynecology. (2016) 215:589.e1–89.e6. doi: 10.1016/j.ajog.2016.05.029, PMID: 27242204

[28] Moreno-Sepulveda J Romeral C Niño G Pérez-Benavente A . The effect of laparoscopic endometrioma surgery on anti-Müllerian hormone: A systematic review of the literature and meta-analysis. JBRA assisted Reprod. (2022) 26:88–104. doi: 10.5935/1518-0557.20210060, PMID: 34755503 PMC8769171

[29] Muzii L Bellati F Bianchi A Palaia I Manci N Zullo MA . Laparoscopic stripping of endometriomas: a randomized trial on different surgical techniques. Part II: pathological results. Hum Reprod (Oxford England). (2005) 20:1987–92. doi: 10.1093/humrep/deh851, PMID: 15860498

[30] Muzii L Bellati F Palaia I Plotti F Manci N Zullo MA . Laparoscopic stripping of endometriomas: a randomized trial on different surgical techniques. Part I: clinical results. Hum Reprod (Oxford England). (2005) 20:1981–6. doi: 10.1093/humrep/dei007, PMID: 15802314

[31] Latif S Khanjani S Saridogan E . Endometriosis and *in vitro* fertilization. Med (Kaunas Lithuania). (2024) 60. doi: 10.3390/medicina60081358, PMID: 39202639 PMC11356404

[32] Exacoustos C Zupi E Amadio A Szabolcs B De Vivo B Marconi D . Laparoscopic removal of endometriomas: sonographic evaluation of residual functioning ovarian tissue. Am J obstetrics gynecology. (2004) 191:68–72. doi: 10.1016/j.ajog.2004.01.010, PMID: 15295344

[33] Reich H Abrao MS . Post-surgical ovarian failure after laparoscopic excision of bilateral endometriomas: is this rare problem preventable? Am J obstetrics gynecology. (2006) 195:339–40. doi: 10.1016/j.ajog.2006.03.088, PMID: 16723107

[34] Bourdon M Peigné M Maignien C de Villardi de Montlaur D Solignac C Darné B . Impact of endometriosis surgery on *in vitro* fertilization/intracytoplasmic sperm injection outcomes: a systematic review and meta-analysis. Reprod Sci (Thousand Oaks Calif.). (2024) 31:1431–55. doi: 10.1007/s43032-023-01421-7, PMID: 38168857

[35] Kitajima M Dolmans MM Donnez O Masuzaki H Soares M Donnez J . Enhanced follicular recruitment and atresia in cortex derived from ovaries with endometriomas. Fertility sterility. (2014) 101:1031–7. doi: 10.1016/j.fertnstert.2013.12.049, PMID: 24502890

[36] Maneschi F Marasá L Incandela S Mazzarese M Zupi E . Ovarian cortex surrounding benign neoplasms: a histologic study. Am J obstetrics gynecology. (1993) 169:388–93. doi: 10.1016/0002-9378(93)90093-x, PMID: 8362952

[37] Muzii L Di Tucci C Di Feliciantonio M Galati G Di Donato V Musella A . Antimüllerian hormone is reduced in the presence of ovarian endometriomas: a systematic review and meta-analysis. Fertility sterility. (2018) 110:932–40.e1. doi: 10.1016/j.fertnstert.2018.06.025, PMID: 30316440

[38] Streuli I de Ziegler D Gayet V Santulli P Bijaoui G de Mouzon J . In women with endometriosis anti-Müllerian hormone levels are decreased only in those with previous endometrioma surgery. Hum Reprod (Oxford England). (2012) 27:3294–303. doi: 10.1093/humrep/des274, PMID: 22821432

[39] Esinler I Bozdag G Arikan I Demir B Yarali H . Endometrioma ≤3 cm in diameter per se does not affect ovarian reserve in intracytoplasmic sperm injection cycles. Gynecologic obstetric Invest. (2012) 74:261–4. doi: 10.1159/000339630, PMID: 22797146

[40] Nieweglowska D Hajdyla-Banas I Pitynski K Banas T Grabowska O Juszczyk G . Age-related trends in anti-Mullerian hormone serum level in women with unilateral and bilateral ovarian endometriomas prior to surgery. Reprod Biol endocrinology: RB&E. (2015) 13:128. doi: 10.1186/s12958-015-0125-x, PMID: 26596960 PMC4657379

[41] Karadağ C Yoldemir T Demircan Karadağ S Turgut A . The effects of endometrioma size and bilaterality on ovarian reserve. J obstetrics gynaecology. (2020) 40:531–36. doi: 10.1080/01443615.2019.1633518, PMID: 31460808

[42] Becker CM Bokor A Heikinheimo O Horne A Jansen F Kiesel L . ESHRE guideline: endometriosis. Hum Reprod Open. (2022) 2022:hoac009. doi: 10.1093/hropen/hoac009, PMID: 35350465 PMC8951218

[43] Vercellini P Somigliana E Viganò P Abbiati A Barbara G Crosignani PG . Surgery for endometriosis-associated infertility: a pragmatic approach. Hum Reprod (Oxford England). (2009) 24:254–69. doi: 10.1093/humrep/den379, PMID: 18948311

[44] Muzii L Marana R Angioli R Bianchi A Cucinella G Vignali M . Histologic analysis of specimens from laparoscopic endometrioma excision performed by different surgeons: does the surgeon matter? Fertility sterility. (2011) 95:2116–9. doi: 10.1016/j.fertnstert.2011.02.034, PMID: 21411079

[45] Paik H Jee BC . Impact of ablation versus cystectomy for endometrioma on ovarian reserve, recurrence, and pregnancy: an updated meta-analysis. Reprod Sci (Thousand Oaks Calif.). (2024) 31:1924–35. doi: 10.1007/s43032-024-01512-z, PMID: 38509401

[46] Ata B Turkgeldi E Seyhan A Urman B . Effect of hemostatic method on ovarian reserve following laparoscopic endometrioma excision; comparison of suture, hemostatic sealant, and bipolar dessication. A systematic review and meta-analysis. J minimally invasive gynecology. (2015) 22:363–72. doi: 10.1016/j.jmig.2014.12.168, PMID: 25573183

[47] Ronsini C Iavarone I Braca E Vastarella MG De Franciscis P Torella M . The efficiency of sclerotherapy for the management of endometrioma: A systematic review and meta-analysis of clinical and fertility outcomes. Med (Kaunas Lithuania). (2023) 59. doi: 10.3390/medicina59091643, PMID: 37763762 PMC10535205

[48] Zhang Y Zhang S Zhao Z Wang C Xu S Wang F . Impact of cystectomy versus ablation for endometrioma on ovarian reserve: a systematic review and meta-analysis. Fertility sterility. (2022) 118:1172–82. doi: 10.1016/j.fertnstert.2022.08.860, PMID: 36334993

