# Correction: Impact of endometriotic cystectomy on ovarian reserve function and ovulation induction outcomes in women with endometriosis undergoing assisted reproductive technology

**DOI:** 10.3389/fendo.2026.1806799

**Published:** 2026-02-24

**Authors:** Yutao Li, Yu Gong, Haiyan Jiang, Meng Ji

**Affiliations:** 1 Department of Assisted Reproduction Center, Sichuan Provincial People’s Hospital, University of Electronic Science and Technology of China, Chengdu, Sichuan, China; 2 Department of Outpatient, Sichuan Provincial People’s Hospital, University of Electronic Science and Technology of China, Chengdu, Sichuan, China

**Keywords:** endometriosis, endometriotic cystectomy, IVF/ICSI, ovarian reserve function, ovulation induction results

The affiliations for all authors were erroneously given as “Sichuan Academy of Medical Sciences and Sichuan Provincial People’s Hospital, Chengdu, China”. The correct affiliations are as listed in the updated author list below: Yutao Li^1^, Yu Gong ^2^, Haiyan Jiang^1^*, Meng Ji^1^*

“1 Department of assisted reproduction center, Sichuan Provincial People’s Hospital, University of Electronic Science and Technology of China, Chengdu 610041, Sichuan, China

2 Department of outpatient, Sichuan Provincial People’s Hospital, University of Electronic Science and Technology of China, Chengdu 610041, Sichuan, China”

The corresponding author information in the published article was incomplete. The correct corresponding author are “Haiyan Jiang (Zenghao5640@126.com) and Meng Ji (wangjiaa0420@sina.com”. The symbol “*” has now been added to their names in the author list to denote equal contribution

Authors “Yutao Li and Yu Gong” were erroneously omitted as equal contributing authors. A symbol has now been added to their names in the author list to denote equal contribution.

The original version of this article has been updated.

